# The potent roles of salt-inducible kinases (SIKs) in metabolic homeostasis and tumorigenesis

**DOI:** 10.1038/s41392-020-00265-w

**Published:** 2020-08-12

**Authors:** Zicheng Sun, Qiwei Jiang, Jie Li, Jianping Guo

**Affiliations:** 1 Institute of Precision Medicine, the First Affiliated Hospital, Sun Yat-Sen University, Guangzhou, Guangdong 510275 China; 2 Department of Breast and Thyroid Surgery, the First Affiliated Hospital, Sun Yat-Sen University, Guangzhou, Guangdong 510275 China

**Keywords:** Drug development, Target identification, Molecular medicine

## Abstract

Salt-inducible kinases (SIKs) belong to AMP-activated protein kinase (AMPK) family, and functions mainly involve in regulating energy response-related physiological processes, such as gluconeogenesis and lipid metabolism. However, compared with another well-established energy-response kinase AMPK, SIK roles in human diseases, especially in diabetes and tumorigenesis, are rarely investigated. Recently, the pilot roles of SIKs in tumorigenesis have begun to attract more attention due to the finding that the tumor suppressor role of LKB1 in non-small-cell lung cancers (NSCLCs) is unexpectedly mediated by the SIK but not AMPK kinases. Thus, here we tend to comprehensively summarize the emerging upstream regulators, downstream substrates, mouse models, clinical relevance, and candidate inhibitors for SIKs, and shed light on SIKs as the potential therapeutic targets for cancer therapies.

## Introduction

Salt-inducible kinase (SIK) was first identified in the adrenal glands of high salt diet-fed rats in 1999.^1^ Further, the SIK family members, including SIK1–SIK3, are characterized as serine/threonine kinases that belong to AMP-activated protein kinase (AMPK) family.^2,3^ Later, SIKs have shown self-phosphorylation, and play an important role in regulating adrenocortical function under the stimulation of high salt or adreno-cortico-tropic-hormone (ACTH).^1^ Of note, the SIK1 is abundantly expressed in the adrenal cortex, as well as in the adipose and neural tissues,^3–5^ while both SIK2 and SIK3 are ubiquitous in humans and mainly expressed in adipose and neural tissues, respectively.^3^ In addition, these SIK family members are dysregulated in various cancers, including ovarian, breast, prostate, and lung cancers, indicating that SIKs may execute crucial roles in tumor occurrence or progression.^3,6^

In recent years, although the roles of SIKs in tumorigenesis have drawn much attention due to their association with TGFβ-Smad, AKT, Hippo, NF-κb and other signaling pathways involved in cancer progression,^6–17^ similar to the AMPK kinases, the potential roles of SIKs in tumorigenesis are still controversial as oncogene or tumor suppressor in a tissue context dependent manner. Therefore, the purpose of this review is to comprehensively summarize the upstream regulators, downstream effectors, clinical relevance, as well as candidate inhibitors of SIKs, to highlight the potential strategy to target SIKs for cancer therapies.

## The upstream regulators and downstream substrates of SIKs

**Fig. 1:**
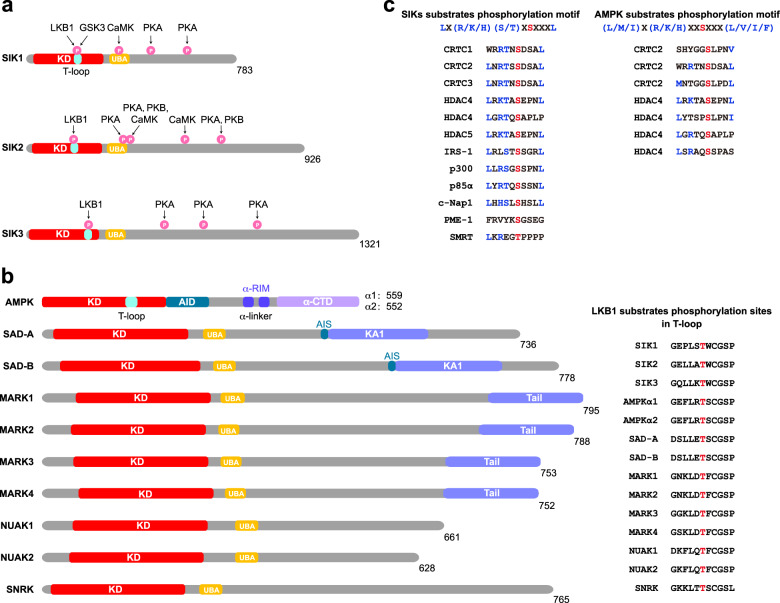
The diagram structure of SIKs and related kinases. **a** The structure and phosphorylation residues are illustrated. SIKs are composed of KD (kinase domain) containing an LKB1 phosphorylation site, SNH domain containing UBA (ubiquitin-associated) domain and C-terminal domain containing multiple PKA phosphorylation sites. **b** The structure of AMPK-related family kinases are illustrated. These kinases share a similar structure with SIKs. AMPKα subunits are composed of KD, AID (autoinhibitory domain), α-linker containing two α-RIM (regulatory subunit-interacting motif) and α-CTD (C-terminal domain). SADs are composed of KD, UBA domain, and KA1 (kinase-associated domain 1), it is N-terminal next to the AIS sequence (autoinhibitory sequence). MARKs are composed of KD, UBA domain, spacer, and tail domain (including the KA1 domain). NUAKs are composed of KD and UBA domain. SNRK is composed of KD and UBA domain. The phosphorylation sites on the T-loop of AMPK-related family kinases are illustrated. The AMPK-related family kinases can be directly phosphorylated and activated by LKB1 on their T-loop (right panel). **c** SIK and AMPK downstream substrate phosphorylations are illustrated. SIKs phosphorylated LX(R/K/H)(S/T)XSXXXL motif (underlined, phosphorylated residue, X, any residue) and the identified AMPK substrates phosphorylation sites reside in the known AMPK phosphorylation consensus sequence (L/M/I)X(R/K/H)XXSXXX(L/V/I/F) are illustrated

*SIK1* gene is located in human chromosome 21, while *SIK2* and *SIK3* genes are both located on chromosome 11.^2^ SIKs share similar structures to AMPK-related kinases, including AMPKα1/α2, SAD-A/B, MARK1–4, NUAK1/2, and SNRK, all of which can be phosphorylated and activated by liver kinase B1 (LKB1). Generally, AMPK-related kinases consist of two common domains, possessing an N-terminal serine-threonine kinase domain (KD) followed by a ubiquitin-associated (UBA) domain.^18–21^ Beyond that, SIKs are also composed of a central sucrose non fermenting (SNF-1) homology (SNH) domain, and a long C-terminal domain (Fig. 1a).^20,22^ The N-terminal KD contains a LKB1 phosphorylation site and is relatively conserved across SIK family. However, the SNH domain is distinct in SIKs, specifically, the similarity percentage of SIK2 and SIK3 compared that of SIK1 is 70% and 37% respectively. The C-terminal domain contains multiple protein kinase A (PKA) phosphorylation sites and is highly conserved between SIK1 and SIK2.^22^ Like other AMPK family members, an activation loop (T-loop) exists in the KD of SIKs, which near the substrate-binding pocket and is phosphorylated and activated by LKB1 (Fig. 1a, b).^19,22^ In addition, there is also an autophosphorylation residue in the T-loop, which is essential for the kinase activity of SIK1 and SIK2.^23^ On the other hand, a UBA domain has also been defined within the SNH domain,^24^ and mutations derived from the UBA domain notably decreased LKB1-mediated SIK phosphorylation and kinase activation,^24^ partially via preventing SIK interacting with 14-3-3 adapter protein to promote SIK nuclear transport.^24,25^ Similar to AMPK kinases, the Thr322 residue in SIK1 SNH domain could also be activated by calcium-dependent protein kinase (CaMK)-mediated phosphorylation,^20,26^ similar results were observed in SIK2 kinase and resulted in SIK2 degradation.^27^ SIKs are considered rapid turnover proteins due to the phosphorylation by PKA, PKC, and tyrosine kinase in their C-terminal region (Fig. 1a).^17,20^ Thus, SIK family members share a similar structure, and play redundant and distinct roles in regulating biological processes, especially in metabolic homeostasis, which will be further summarized in the following sections.

### SIK upstream regulators

Acting as AMPK-related kinases, SIKs exhibit a similar activation property with AMPK,^19,22^ in an LKB1-mediated phosphorylation dependent manner (Fig. 1a, b).^18–20,28^ Importantly, physiological changes, such as energy deprivation, insulin, or glucagon perturbation, all manipulate SIK kinase activity.^29^ For example, insulin stimulation or chronic hyperglycemia could increase SIK protein level and kinase activity.^30–32^ By contrast, Patel et al.^33^ reported that insulin did not regulate SIK2 phosphorylation and activity. Different from other AMPK-related family members, SIKs could be specifically activated by the sodium homeostasis.^2^ As a result, sodium intake-induced calcium influx affected by Na^+^/Ca^2+^ exchange system (NCE1), could cause CaMK-mediated SIK1 phosphorylation and activation,^26,34,35^ which was argued by another study.^36^

#### Liver kinase B1

LKB1 protein kinase was initially identified in *Peutz Jeghers* syndrome (PJS),^37^ and later it has been considered a master serine/threonine kinase involved in diverse physiological processes.^38^ Accumulating evidence has demonstrated that LKB1 can phosphorylate and activate many AMPK-related kinases on their T-loop (Fig. 1b).^18–21^ Genetically, deletions of *LBK1* are frequently occurred in NSCLCs, especially in KRAS^G12D^-bearing NSCLCs,^39,40^ indicating that *LBK1* is a potent tumor suppressor gene. Although previous efforts mainly devoted to the studies of AMPK roles in LBK1 tumor suppressor functions, recently, depletion of *AMPKα1* or *AMPKα2* could not markedly impair LKB1 tumor suppressive roles in KRAS^G12D^-driven NSCLC models,^41^ indicating that other substrates will play more important roles in mediating LBK1 tumor suppressor functions. As such, SIK1 and SIK3 have been revealed as the predominant downstream targets of LKB1 in mediating anti-tumorigenesis effect in NSCLC.^42,43^ While some studies provided that SIK2 underwent autophosphorylation and activation in vitro independent on the presence of LKB1.^7^ Therefore, whether other members of AMPK subfamily mediating LBK1 functions in metabolic homeostasis and tumorigenesis need more investigations, especially in combination with their conditional KO mouse models.

#### Ca^2+^–CaMK

Ca^2+^–CaMK is another important upstream regulator of SIKs, in an LKB1 independent manner.^7,44^ In the absence of LKB1, there is still a residual activation of SIK1, which may be due to the activation by CaMK.^42,43^ Phospholipase C (PLC) can boost Ca^2+^ influx from endoplasmic reticulum (ER) to the cytoplasm via inositol triphosphate (IP3) receptor, thus activating the CaMK, which leads to the phosphorylation and activation of SIK2 at Ser358.^7,45^ PKA can also phosphorylate SIK2 at Ser358.^7,20^ But PKA is not involved in PLC-mediated SIK2 phosphorylation at Ser358 and activation.^7^ As discussed earlier, sodium mediated SIK1 activation is also through CaMK.^26,34,35^ Interestingly, a study found that CaMK I/IV phosphorylated SIK2 at Thr484, leading to SIK2 degradation and promoting CREB-mediated transcription (Fig. 1a).^27^

#### Protein kinase A

PKA, one of the members of AGC kinase, is a tetrameric holoenzyme composed of homodimer including two kinds of regulatory subunits (RIα and RIβ, RIIα and RIIβ) and three catalytic subunits (Cα, Cβ, or Cγ).^46,47^ PKA activity depends on the binding of cAMP with the regulatory subunits, leading to the release of active catalytic subunits and then phosphorylating diverse substrates.^46^ Pathologically, mutations in *PKA* catalytic subunit promoted adrenal cortical tumorigenesis and *Cushing’s* syndrome.^48,49^ Although PKA is not considered as an oncogene, PKA has an active role in several cancers,^50–52^ and induce the transformation of human mammary stromal cells into epithelial cells (MET).^53^ Until now, all three SIK family members have been discovered to undergo PKA-mediated phosphorylation and inhibition. Bioinformatic analyses imply that SIKs contain multiple motifs harboring PKA phosphorylation and 14-3-3 binding sites (RSXSXP; underlined, phosphorylated residue; X, any residue).^20,29,54^ When these potential phosphorylation residues are abolished, the binding of SIKs with 14-3-3 is largely eliminated, which markedly antagonizes PKA inhibitory roles on SIKs.^29,54^ Notably, changes in these residues do not affect LKB1-mediated SIKs activation. Biologically, PKA can phosphorylate SIK1 to promote its nucleus translocation,^55–57^ which could be efficiently blocked by mutating these two arginine residues within the phosphorylation motif.^56^ Similarly, PKA directly phosphorylates SIK2 to regulate its stabilization and relocation by modulating its interaction with 14-3-3.^7,58^ Meanwhile, the deletion of *PKA* not only promotes SIK1 protein stability, but also transcriptionally accelerates *SIK1* expression.^59^ Hence, PKA would be a critical negative upstream regulator of SIKs, to compete with LKB1 in governing SIK physiological or pathological functions.

#### Other upstream regulators

Aside from phosphorylation, other post-translational modifications (PTMs), such as acetylation, also play important roles in governing SIK activity. Of note, p300-mediated acetylation inhibited ATP binding with and activating of SIK2 by disturbing its phosphorylation at Thr175, conversely, SIK2 can also directly phosphorylate and regulate p300 acetyltransferase.^60^ In addition, HDAC6 has been identified to activate SIK2 by removing its acetylation modification.^9^ In addition, RNF2, an E3 ligase, has been revealed to ubiquitinate and in turn degrade SIK1 in hepatoma cells.^61^ Consequently, the specific regulatory mechanisms of other PTMs to SIKs need to be further explored for fully understanding the upstream regulation for SIKs.

### SIK downstream substrates

Similar to AMPK in recognizing the substrate motif (L/M/I)X(R/K/H)XXSXXX(L/V/I/F),^62,63^ SIKs phosphorylates substrates containing LX(R/K/H)(S/T)XSXXXL motif (Fig. 1c).^54,55^ A variety of metabolic regulators, including CRTC and class IIa HDACs, are common substrates of both AMPK and SIKs.^29^ Importantly, SIKs, but AMPKs, can directly phosphorylate some specific substrates, including CRTC/CREB and PPase methylesterase-1 (PME-1) to involve in metabolic homeostasis.^34^

#### HDAC

Histone deacetylases functionally remove the acetylation modification from both histone and nonhistone proteins.^64^ Among the histone deacetylases, class IIa HDACs (HDAC4, 5, 7, and 9) are inhibitors of different transcription factors, especially for MEF2 family.^64^ All three SIK family members have emerged as new kinases for class IIa HDACs.^57,65^ SIK-mediated HDAC phosphorylation promotes its binding with 14-3-3, and facilitates its transport from nucleus to cytoplasm, and then represses MEF2-dependent transcription.^57,65^ Therefore, SIKs can regulate the development of skeletal muscle, skeleton, regulatory T cells as well as leukemia and other pathological processes via manipulating class IIa HDACs as discussed above.^29,57,59,64,66^

#### CREB-regulated transcription co-activator/cAMP response element-binding protein

cAMP response element-binding protein (CREB) and CREB-regulated transcription co-activator (CRTC) affect cell proliferation, differentiation, metabolism, and other biological processes.^67^ Increased CREB activity confers to tumor progression, chemotherapy resistance, and reduced survival.^68^ CREB is another well-established SIK downstream effector. Although SIKs could not directly phosphorylate CREB, they can inhibit CREB in a kinase-dependent manner.^69^ CRTC is a co-activator of CREB, including CRTC1–3, and favors to stabilize CREB or directly contacts with CREB promoters.^70^ CRTC is also helpful for the recruitment of histone acetyltransferase p300 for CREB transcriptional activity. SIKs can directly phosphorylate CRTC, block its association with 14-3-3, and inhibit its nuclear transport, where CRTC binds and enhances CREB driven gene transcription.^8,20,31,32,71^ SIKs also promote COP1-mediated CRTC1 ubiquitination and degradation by phosphorylating its multiple residues.^67^ In keeping with these findings, SIK2 could abrogate CRTC2 acetylation by phosphorylating p300 and integrate with the phosphorylation of CRTC2 to facilitate COP1-mediated CRTC2 ubiquitination and subsequent degradation.^32,72^ Of note, CREB could transcriptionally boost the expression of *Sik1* by binding its enhancer in mouse skeletal muscle cells.^57^ Therefore, it is possible that there is a negative feedback loop between SIKs and CRTC–CREB signaling pathway to influence cellular malignancies.

#### PME-1/Na^+^, K^+^-ATPase

The Na^+^,K^+^-ATPase is widely distributed on the cell membrane, and functions to transport sodium and potassium ions and maintain the balance of osmotic pressure.^73^ The activated SIK1 phosphorylates PME-1, causing its dissociation from the complex of PP2A/PME-1/ Na^+^,K^+^-ATPase,^34^ as a result, PP2A dephosphorylates Na^+^,K^+^-ATPase and attains its catalytic activity.^34^ On the other hand, SIKs also transcriptionally regulate the Na^+^,K^+^-ATPase, either by directly inhibiting the entry of CRTC into the nucleus to transcript *ATP1B1* gene, which encodes a Na^+^,K^+^-ATPase subunit,^2^ or by indirectly repressing the hormones-induced Na^+^,K^+^-ATPase expression,^74^ via an increased *CYP11A* and *StAR* mRNA levels to promote the adrenocorticotropic hormone production.^55,75–77^

### Other downstream signaling pathways

#### TGFβ-Smad pathway

In normal epithelial cells, TGFβ-Smad signaling pathway is considered to play an anti-cancer role by inducing cell cycle arrest and apoptosis.^78^ However, during the late stage of tumorigenesis, TGFβ-Smad promotes cancer cell EMT and plays a pilot role in promoting cancer.^79^ *SIK1* is considered as a transcriptional substrate of TGFβ-Smad pathway,^80^ meanwhile, activated SIK1 may regulate the contraction phenotype of vascular smooth muscle cells by inhibiting TGFβ1 signaling to prevent high salt intake-caused hypertension.^81^ Recent studies also indicate that SIKs function as a negative feedback in the TGF-β signal by formatting the SIK1–Smad7–SMURF2 (SMAD-specific E3 ubiquitin protein ligase 2) complex, to ubiquitinate ALK5 to repress TGFβ signaling pathway.^80,82^ Notably, high glucose-mediated downregulation of SIKs results in the stabilization of ALK5 in mesangial cells.^83^ Furthermore, one study suggested that SIK1 phosphorylated polarity protein partitioning-defective 3 (Par3) to promote its degradation via both proteasome and lysosome manners.^84^ A recent research also revealed that SIK inhibitors could repress the TGF-β-mediated transcriptional capability of plasminogen activator inhibitor 1 (PAI-1) and cellular apoptosis without affecting the phosphorylation or nuclear translocation of R–Smads complex.^85^ Of note, this might be via SIK1, but not SIK2 or SIK3, to control Smad-associated transcriptional cofactors via phosphorylating CRTC.^85^

#### Hippo pathway

The Hippo signaling pathway was conservative and initially identified in *drosophila*, which plays a major role in controlling organ size.^86^ SIK2 and SIK3 have been proved to be upstream regulators of the Hippo signaling pathway in *drosophila*. Mechanistically, they can directly phosphorylate the scaffold protein Salvador (Sav), a core component of Hippo complex, to prevent the oncogene driven inhibition of Yki, an ortholog of Yes-associated protein (YAP).^87^ As an important hub of Hippo signaling pathway, YAP activation leads to the inhibition of cell contact and facilitates tumor cell metastasis.^86,88^ Specifically, SIK2 can directly trigger Yki/YAP transcription activity to increase the Yki/YAP target gene expression and promote tissue overgrowth,^87^ indicating the potential oncogenic role of SIK2 in tumorigenesis.

#### NF-κb signaling pathway

**Fig. 2:**
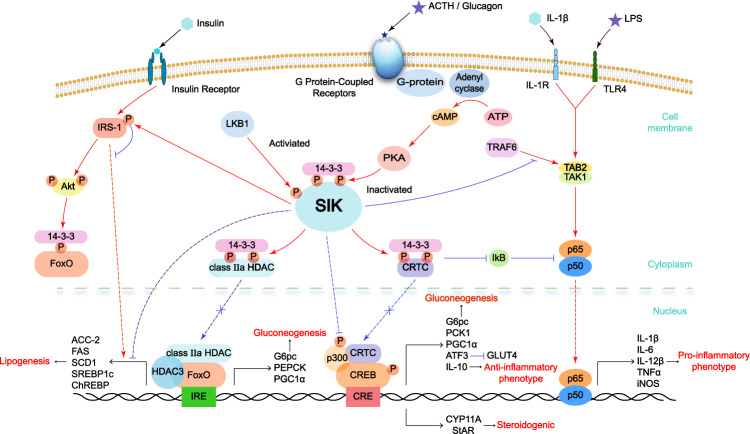
The roles of SIKs in the regulation of glucose, lipid metabolism and inflammation are illustrated. SIKs’ regulatory effect is mainly through phosphorylating CRTC and class IIa HDACs to retain them in the cytoplasm. Therefore, SIKs repress various gene expression and then inhibit gluconeogenesis, lipogenesis, steroidogenesis and the production of IL-10. Besides, SIK2 upregulates GLUT4 expression by inhibiting transcriptional repressor ATF3, leading to glucose uptake. SIKs promote NF-κb signaling pathway and production of inflammatory factors such as IL-1β, IL-6, IL-12β, TNFα, and iNOS through downstream substrates CRTC. However, SIK1 and SIK3 inhibit the interaction of TRAF6 and TAB2, and then repress NF-κb signaling pathway. Energy deprivation and hormone (insulin, glucagon, and ACTH) presence all control the activity of SIKs kinase and regulate their effect on metabolism

NF-κb signaling pathway is one of the well-established inflammatory pathways, by which SIKs could manipulate the production of inflammatory factors (Fig. 2). Meanwhile, CRTC and class IIa HDACs, two important downstream substrates of SIKs, negatively regulate NF-κb signaling pathway.^89–91^ However, it is noteworthy that the effects of SIK1 and SIK3 on NF-κb signaling pathway seem to be distinct, they prefer to inhibit the binding of TAB2/TRAF6 to repress the NF-κb signal.^92^

#### PI3K-AKT signaling pathway

To date, the correlation between SIKs and AKT signaling pathway is focused on SIK2 (Fig. 4).^7,10,11,30,93^ SIK2 leads to a decrease of AKT phosphorylation, which may be due to the SIK2-mediated IRS-1 phosphorylation, thus inhibiting the insulin signaling pathway.^30^ However, in tumor cells, the effect of SIK2 on the AKT signaling pathway seems to be changed to elevating PI3K/AKT activity.^7,10,11,93^ Mechanistically, SIK2 can directly phosphorylate p85, a regulatory subunit of PI3K complex, to activate the AKT kinase activity.^7^ As a result, SIK inhibitors, such as ARN-3236, can efficiently reduce AKT phosphorylation and activation in ovarian cancer cells.^10^ However, there is no compelling evidence proven that SIK2 could directly bind and regulate AKT kinase activity,^7,10,11,93^ therefore, the direct connection between SIKs, especially the SIK1 and SIK3, and AKT need to be further explored.

## Biological functions of SIKs in metabolic homeostasis

### SIK functions in gluconeogenesis

Gluconeogenesis is a biological process in which noncarbohydrate precursors (including lactic acid, glycerin, amino acids, etc.) are transformed into carbohydrates (including glucose or glycogen), which can be manipulated by insulin and glucagon controlled the expression of glucose-6-phosphatase (G6Pase) and phosphoenolpyruvate carboxykinase (PEPCK).^29,31,94^ Specifically, PEPCK and G6Pase control the initial and final steps of gluconeogenesis respectively.^95^ Proliferator-activated receptor gamma co-activator (PGC-1α), a direct target of CREB, can largely elevate the expression of PEPCK and G6Pase.^96^ Meanwhile, PGC-1α is also associated with histone acetyltransferase (HAT) p300,^97^ and serves as a key regulator in liver gluconeogenesis and the focal target of cAMP/PKA/CREB axis.^98^ On the other hand, insulin can block the effect of PGC-1α and interfere with the activation of gluconeogenesis through AKT-mediated forkhead box 1 (FoxO1) phosphorylation.^99^ Furthermore, insulin can also regulate the activity of PGC-1α by governing its acetylation and phosphorylation.^100,101^

SIKs and their substrates, such as CRTC and class IIa HDAC, are largely involved in gluconeogenesis (Fig. 2).^20,29^ SIK1 was first found to inhibit gluconeogenesis in the hepatocytes, and its mRNA and protein levels under fasting conditions increased fourfold relative to feeding conditions.^31^ Moreover, overexpression of SIK1 in primary hepatocytes suppressed forskolin or cAMP induced an increase in *Pck1* gene expression via phosphorylation of CRTC2.^31^ Subsequent studies have proven that SIK2 and SIK3 have a similar effect.^32,33,102^ Dentin et al. reported that SIK2 is a downstream substrate of PI3K-AKT signaling pathway response to insulin, subsequently followed by CRTC2 phosphorylation.^32^ Itoh et al.^102^ showed that *SIK3* knockout in hepatocytes was associated with elevated mRNA of *Pgc1a*, *Pepck*, and *G6pc* gene. Collectively, all three SIK isoforms can inhibit gluconeogenesis possibly via SIK-mediated CRTC phosphorylation and restriction in the cytoplasm.^20,54^ CRTC played a key role in gluconeogenic by binding CREB to transcriptionally promote gluconeogenic genes expression, such as *G6PC*, *PEPCK1*, and *PGC-1α* gene.^29,103^ By contrast, in the case of starvation, glucagon can also inhibit the catalytic activity of SIKs via PKA-mediated phosphorylation and facilitate gluconeogenesis.^29^ On the other hand, SIKs directly phosphorylated class IIa HDACs to block their nuclear translocation^20,29^ and interaction with FoxO1 on *PEPCK* and *G6Pase* promoter regions, thereby stimulating gluconeogenesis.^104^ Conceivably, SIK inhibitors could compromise the phosphorylation of CRTC2/3 and HDAC4/5, leading to gluconeogenic gene expression and glucose production.^20,33,54^ As such, loss-of-function mutations of *SIKs* or deficiency of *LKB1* could efficiently antagonize gluconeogenesis.^20,29,33^

While SIKs have markedly involved in diverse signaling pathways to regulate gluconeogenesis, several studies demonstrated that SIK1 and SIK2 did not impact gluconeogenesis alone in mouse model.^33,105^ Of note, in liver specific *Sik1* and *Sik2* double KO mice, CRTC phosphorylation and gluconeogenesis were not influenced,^33,105^ instead, *SIK3* plays a key role in regulating gluconeogenesis rather than *SIK1* and *SIK2*.^102^ Under the conditions of lactate-induced gluconeogenesis, the blood glucose level of *Sik3*, but not *Sik1* and *Sik2*, KO mice were rapidly increased than that of WT mice, indicating that Sik3 plays an important role in mouse gluconeogenesis.^66,102^ Though SIKs display a controversial role in gluconeogenesis, it is generally accepted that SIKs can reduce insulin sensitivity and promote energy storage by inhibiting gluconeogenesis.

### SIK functions in glucose uptake

The process of glucose uptake mainly depends on the expression of sodium-dependent glucose transporter and glucose transporter (GLUT).^106^ The majority of peripheral glucose uptake in adipose tissue and skeletal muscle are achieved by insulin-responsive glucose transporter 4 (GLUT4).^106^ Importantly, GLUT4 expression has been negatively regulated by various upstream regulators, including but not limited to HDAC4, CRTC2/3, and protein phosphatase 2A (PP2A).^107^ These proteins are all well-established SIK2 downstream substrates, indicating that SIK2 is a positive regulator of glucose intake by upregulating GLUT4 expression (Fig. 2).^107–109^ Meanwhile, CREB upregulated the expression of transcriptional repressor activating transcription factor 3 (ATF3), and thereby downregulated the GLUT4, resulting in promoting insulin resistance.^107,110^ Consistently, inactivating SIK pharmacologically or genetically could reduce GLUT4 expression and glucose uptake.^107–109^ However, SIK1 promotes insulin resistance and inhibits glucose uptake in skeletal muscle possibly via directly phosphorylating insulin receptor substrate 1 (IRS-1).^105,111^ *Sik1* KO did not lead to hyperglycemia and gluconeogenesis in vivo, but significantly improved glucose tolerance, peripheral insulin sensitivity, and skeletal muscle glucose uptake upon high-fat diet due to elevated expression of GLUT4, GLUT1, and GLUT12.^105^

### SIK functions in lipid metabolism

In addition to its role in glucose metabolism, SIKs also seems to function as a negative regulator of lipid metabolism (Fig. 2). Lipid is an important source of energy and substance for cell homeostasis, and its metabolic process is tightly regulated by a complex network.^112^ The fatty acid, a vital and raw material for triglycerides,^112^ is synthesized mainly by two key enzymes, acetyl-CoA carboxylase (ACC) and fatty acid synthase (FAS).^112^ SIK1 represses lipogenic gene expression such as *Acaca* (acetyl-CoA carboxylase), *Fasn* (FAS), *Srebf1* (sterol regulatory element-binding transcription factor 1) and *Scd1* (stearoyl-CoA desaturase-1), possibly via an SREBPs (sterol regulatory element-binding protein)-mediated transcriptional regulation.^113^ Overexpression of SIK1 in hepatocytes induced high mRNA levels of the lipogenic gene (*Srebf1*, *Fasn*, and *Scd1*) and high protein levels of ACC and FAS.^113^ SREBP-1c is directly phosphorylated by SIK1 at Ser329, which is proposed to be required for SIK1 in repressing lipogenic gene expression.^113^ Steroids are another kind of lipid, including estrogen, progesterone, and adrenocorticotropic hormone.^77,114^ Steroidogenic acute regulatory protein (StAR) and cytochrome P450 cholesterol side chain cleavage (P450scc) are two key enzymes in steroidogenesis.^77,114^ StAR regulates the transport of cholesterol from the outer membrane to the inner membrane in mitochondria, which is the key rate-limiting step of steroid synthesis.^114^ In addition, *CYP11A* gene encodes P450scc, a cholesterol side chain cleavage enzyme that catalyzes the conversion of cholesterol to pregnenolone, a precursor of steroid.^77^ SIK1 plays a key role in steroidogenesis and adipogenesis mediated by governing ACTH signaling pathway.^22,55,115^ The mRNA levels of SIK1 in mouse adrenal cortex cells (Y1 cells) stimulated by ACTH peaked rapidly within 1 h, then decreased gradually, and returned to the basic level after 12 h. However, the mRNA levels of P450scc and StAR began to rise after a few hours, reaching the highest levels after 8 h.^115^ The transcription of SIK1 occurred before the ACTH stimulated StAR and P450scc transcription, so it can be speculated that SIK1 is associated with steroidogenic gene expression.^115^ On the other hand, SIK1 overexpression significantly repressed the ACTH-dependent expression of P450scc and StAR in Y1 cells.^115^ Subsequent studies demonstrated that SIK1 repressed the efficient operation of the CREB transcription activation complex, thereby inhibiting the CRE-driven transcription of the *CYP11A* gene and the *StAR* gene in Y1 cells.^55,75–77^

Du et al.^116^ found that, similar to SIK1, SIK2 can also repress the expression of lipogenic genes (*FAS*, *ACC2* and *SCD1*), and this effect can be reversed by depleting *SREBP1*. In addition, SIK2 promotes fatty acid synthesis by upregulating SREBP1c expression, thus promoting the transcription of *Fasn* in ovarian cancer cells.^93^ Meantime, SIK2 also promotes cholesterol synthesis by upregulating SREBP2 expression, to transcriptionally elevate cholesterol synthetase, 3-hydroxy-3-methyl-glutaryl-coenzyme A reductase (HMGCR).^93^ Importantly, SIK2 phosphorylated and inhibited p300 activity, leading to the decreased acetylation of carbohydrate response element-binding protein (ChREBP), which plays a positive role in lipogenic and gluconeogenesis.^60^ SIK2 also phosphorylated IRS-1 to attenuate insulin driven lipogenesis in human adipocytes.^55^ Another study also showed depletion of *SIK2* promoted increased adipogenic potential and insulin resistance in preadipocytes in a CRTC2-dependent manner.^110^ SIK2 controlled FAO in liver and skeletal muscle, as such, *Sik2* KO mice displayed the decreased key enzymes in the process of FAO, such as carnitine carnitine palmitoyl-transferase 1 (CPT-1), mitochondrial medium chain acyl COA dehydrogenase (MCAD), and peroxisomal acyl-CoA oxidase (ACOX1).^110^ Inconsistently, SIK2 promotes FAO by phosphorylating ACC1 and inhibiting CPT1A in ovarian cancer cells, resulting in promoting abdominal metastasis.^7^ In addition, some studies showed that *Sik2* KO mice do not impact lipid metabolism in vivo.^33^

SIK3 has been reported as a new energy regulator by promoting lipid storage in *Drosophila* through compromising the activity of HDAC4 and CRTC.^20,117^ SIK3 also regulated cholesterol and bile acid metabolism by combining with retinoic acid metabolism and might alter energy storage in mice.^118^ Inhibition of fatty acid synthesis was observed in *Sik3* KO mice,^118^ however, the roles of SIK3 in regulating lipid metabolism are not good evaluated in mammal animals.^20^ Based on these observations, although SIKs have been considered to play important roles in lipid metabolism, the mechanism of SIKs regulating lipid metabolism has not been well elucidated yet. Thus, more efforts are desired in the future to explore the diverse and distinct roles of SIK family members in lipid metabolic homeostasis.

### SIK functions in inflammation

Inflammation is an important pathological change tightly related to tumorigenesis. Inflammation predominantly changes the tumor microenvironment and accelerates tumor occurrence, growth, and metastasis.^119^ An important aspect of controlling inflammation is reprogramming macrophages, to promote transformation from classic activated macrophage (M1 macrophage) to regulatory macrophage (M2 macrophage).^120^ Of note, SIKs act as molecular switches in regulating M1–M2 macrophage transformation (Fig. 2).^8,71,120^ The observation that SIK inhibitors compromised CRTC3 phosphorylation in TLR-stimulated macrophages, led to increased CREB-dependent gene expression, including *IL-10*, and reduced pro-inflammatory cytokine expression, such as *TNFα* and *IL-6*.^8,71^ Importantly, IL-10 drives an anti-inflammatory function by promoting the expression of regulatory M2b macrophage markers, such as SPHK1, LIGHT, and Arg1.^8,120^ Similar results were also observed in dendritic cells (DCs).^71^ On the other hand, SIK inhibitors decreased the production of pro-inflammatory cytokines, but not IL-10 in IL-1β-mediated macrophages, possibly due to the insufficient CRTC3 phosphorylation.^71^ Moreover, other upstream regulators, for example, prostaglandin E2 (PGE2), also induced IL-10 production via the PKA–SIK–CRTC signaling pathway in the quiescent myeloid cells.^29,121^ Consistently, SIK inhibitors can mediate the anti-inflammatory phenotype through activating NF-κb pathway. Briefly, the non-phosphorylated form of CRTC could increase CREB activity, which upregulated the expression of IκB to repress the NF-κb mediated inflammatory response.^91^ Meanwhile, in macrophages, inhibition of SIK pharmacologically or genetically repressed HDAC4 phosphorylation and abrogated its roles in deacetylating NF-κb subunit p65, resulting in reduced TNFα and IL-12β expression.^90^

While SIK inhibitors have a clear anti-inflammatory effect, whether different SIK family members play the same role in inflammatory response remains controversial. Of note, SIK3 is considered as a negative regulator of inflammatory cytokines such as IL-6, nitric oxide (NO), and IL-12 in macrophages. The pro-inflammatory cytokines expression level was increased and the LPS-induced endotoxic shock was aggravated in *Sik3*-, rather than in *Sik1*-, and *Sik2*-KO mice. As a result, *Sik3* KO mice died within 48 h after LPS injection.^89^ Some evidence also suggest that SIK1 and SIK3 can inhibit toll-like receptor (TLR) signal not only through IKK-mediated NF-κb signaling pathway, but also through TGFβ-activated kinase 1-binding protein 2 (TAB2)-tumor necrosis factor receptor-associated factor 6 (TRAF6) complex.^89,92^ SIK1 and SIK3 inhibit the binding of TAB2 and TRAF6 to regress the NF-κb pathway, and then affect the production of pro-inflammatory cytokines.^92^ These results together show that SIK1 or SIK3 may play an important role in promoting the anti-inflammatory phenotypes, which is opposite to the function of SIK2.^91,92^

In summary, the primary mechanism of SIKs in altering inflammatory factors is through phosphorylating CRTC and regulating the NF-κb signaling pathway.^8,71^ Compared with broad-spectrum immunosuppressants such as glucocorticoids, SIK inhibitors may have more advantages due to a combined effect on anti-inflammatory cytokines.^8,122^ Therefore, SIKs are regarded as therapeutic targets for inflammatory diseases.

### SIK functions in other physiological processes

In addition to the metabolic roles we discussed above, SIKs can also control melanin and bone metabolism. The alpha-melanocyte stimulating hormone (α-MSH) increased secretion upon UV irradiation exploration and could bind melanocortin 1 receptor (MC1R) on the melanocyte membrane to activate adenylate cyclase, resulting in increased intracellular cAMP levels.^123^ Consequently, activated PKA can directly phosphorylate CREB to initiate the transcription cascade of melanogenesis programs, for example, promoting the microphthalmia-associated transcription factor (MITF) expression.^123,124^ Moreover, tyrosinase, induced by MITF, promoted the synthesis of melanin.^125^ More importantly, PKA could also directly phosphorylate SIK2 to control α-MSH/cAMP/CREB axis via inhibiting CRTC1, in which SIK2 acts as a negative regulator in the synthesis of melanin,^125,126^ therefore, the A(y)/a mice with *Sik2* KO show brown hair.^126^

SIK1 is a key negative regulator of osteoblast proliferation and differentiation. The inhibition of SIK1 is of great importance to the osteogenesis of bone morphogenetic protein 2 (BMP2) signal transduction. In osteoblasts, SIK1 regulates bone anabolism through the CRTC1–CREB-Id1 (inhibitor of DNA binding 1) axis. Under the conditions of SIK1 inhibition, non-phosphorylated CRTC1 translocated to the nucleus, stimulating the activity of CREB to induce the expression of osteogenic genes, including *Id1*.^127^ HG-9-91-01, a pan-SIK inhibitor, significantly down-regulated c-Fos and nuclear factor of activated T-cell 1 (NFATc1) protein levels to inhibit osteoclast formation by reducing osteoclast fraction and bone resorption activity.^128^ Another study demonstrated that SIK inhibitor acted a role like PTH, targeting sclerostin (SOST) and receptor activator of NF-κB ligand (RANKL), which are responsible for increasing the ability of bone formation and absorption.^129^ By contrast, SIK3 shows necessary roles for mouse skeletal development, as a result, *Sik3* KO mice show severe skeletal deformities.^127^

### SIK-related mouse models

*Sik1* KO mice displayed significant abnormalities in carbohydrate and lipid metabolism (Table 1). For example, *Sik1* KO mice, generated from *GDF9-Cre*-mediated *Sik1* germline global knockout, exhibited normal blood glucose expression and increasing insulin sensitivity on a high-fat diet.^105^ Similarly, *Sik1* KO mice, generated from *Sik1* KO ES cells, displayed an increased glucose tolerance due to elevated insulin secretion from pancreatic β-cells.^130^ Since *Sik1* global KO mice could not specifically explain the effect of *Sik1* on glucose metabolism in different tissues, *Mark et al*. therefore constructed tissue specific *Sik1* KO mice.^105^ They injected adeno-associated virus (AAV) that expresses *Cre* from the hepatocyte-specific thyroxine-binding globulin (TBG) promoter into *Sik1*^*fl/fl*^ mice, resulting in liver specific *Sik1* 75% deletion. They were surprised to find that liver specific *Sik1* KO could not increase gluconeogenesis.^105^ In addition, they constructed *Sik1*^*fl/fl*^;*Myf5*^*Cre/+*^ mice for skeletal muscle specific *Sik1* KO, in which the insulin sensitivity and glucose uptake were markedly enhanced.^105^

**Table 1:** The summary of SIK mouse models

SIK members	Mouse model types	Functional characteristics	References
SIK1	*Sik1*^*fl/fl*^;*GDF9-Cre* mice *Sik1*^*fl/fl*^;*TBG-Cre* mice *Sik1*^*fl/fl*^;*Myf5*^*Cre/+*^ mice	Abnormal glucose metabolism	^105^
SIK1	*Sik1*^−/−^ mice (generated from *Sik1* KO ES cells)	Elevated insulin secretion and more osteogenic potential	^123,130^
SIK1	*KSik1* (*Kras*^LSLG12D/+^;*R26*^LSL;luc/luc^;*Sik1*^fl/fl^) mice	Increased tumor size and burden	^42^
SIK1	*KT*;*H11*^*LSL-Cas9*^ (*Kras*^*LSL-G12D/+*^;*R26*^*LSL-Tomato*^*;H11*^*LSL-Cas9*^) mice	Increased tumor size	^43^
SIK1	*Sik1*^−/−^ mice	High blood pressure	^81^
SIK2	*Sik2*^−/−^ mice (*Sik2*^*lacZ*^)	Hyperglycemia and hypertriglyceridemia	^110^
SIK2	*Sik2*^*fl/fl*^;*Cre*^+/−^ mice	normal glycemia	^33^
SIK2	*Sik2*^−/−^ mice (generated from *Sik2* KO ES cells)	Enhanced neuronal survival	^27^
SIK2	*Sik2*^−/−^ mice (generated from *Sik2* KO ES cells)	Preventing left ventricular hypertrophy	^134^
SIK3	*Sik3*^−/−^ mice (generated from *Sik3* KO ES cells)	Dystrophic, including lipodystrophy, hypolipidemia, hypoglycemia and hyperinsulinemia, with cholestasis and cholelithiasis phenotype	^118^
SIK3	*Sik3*^−/−^ mice (generated from *Sik3* KO ES cells)	Dwarfism and skeletal deformities	^66^
SIK3	*Sik3*^*fl/fl*^;*Col11α2-11EnhCre* and *Col11α2-ERCre* mice	Achondroplasia and resistance to the osteoarthritis	^131,132^
SIK3	*Sik3*^−/−^ mice (generated from *Sik3* KO ES cells)	Abnormal circadian rhythms	^135^
SIK3	*Sik3*^−/−^ mice (generated from *Sik3* KO ES cells)	Pro-inflammatory phenotype	^89^
SIK3	SIK1-T182A, SIK2-T175A, and SIK3-T163A single and double KI mice (created by ES cells gene targeting technologies)	Anti-inflammatory phenotype	^133^

Nevertheless, *Sik2* KO mice showed abnormalities of hyperglycemia and hypertriglyceridemia, which are related to the glucose absorption and insulin tolerance, increased leukocytes lipolysis, and decreased fatty acid intake in peripheral tissues.^110^ Liver specific *Sik2* KO mice (*Sik2*^*fl/fl*^;*Cre*^+/−^) displayed normal glycemia.^33^ However, *Sik3* KO mice derived from *Sik3* KO embryonic stem (ES) cells exhibited a dystrophic phenotype, including lipodystrophy, hypolipidemia, hypoglycemia, and hyperinsulinemia, with the phenomena of cholestasis and cholelithiasis. Of note, deficient *Sik3*-induced hypoglycemia may be due to the lack of energy storage and the subsequent enhancement of insulin response, similarly, deficient *Sik3*-induced fatty dystrophy phenotype may be related to the inhibition of fatty acid synthesis in the liver and high energy consumption rate.^118^

*Sik3* KO mice showed dwarfism in the process of growth with a minor impact on embryo development.^66^ Anatomic and histological analyses showed that the growth plate and articular cartilage area of the limbs were obviously expanded, the chondrocytes of sternum, rib, and spine were accumulated, and the skull was damaged under the condition of *Sik3* deletion.^66^ Meanwhile, *Sik3* KO mice suffered from severe skeletal deformities, and most of them died at the period of birth.^66^ In order to exclude the systemic changes caused by metabolic abnormalities of other organs in *Sik3* KO mice, *Sik3* chondrocyte conditional KO (*Sik3*^*fl/fl*^*;Col11a2-11EnhCre*) mice have been generated and showed a phenotype of achondroplasia, such as dwarfism with a similar histological change on the formation of endochondral bone.^131^ More importantly, the conditional KO of chondrocyte *Sik3* (*Sik3*^*fl/fl*^*;Col11a2-ERCre*) resulted in the thickening of articular cartilage in adult mice, leading to resistant to the osteoarthritis, a phenotype related to the decrease of type X collagen (COL10) expression in the noncalcified area of articular cartilage.^132^ Consistently, *Sik1* KO mice derived from *Sik1* KO ES cells displayed a similar enhancement of osteogenic ability with higher bone mass, osteoblast number, and bone formation rate compared with WT mice.^127^ In keeping with this finding, the osteoblasts derived from *Sik1*, but not *Sik3* KO mice, exhibited more osteogenic possibility than cells derived from counterpart mice, such as increased differentiation of osteoblasts and mineralization of bone matrix.^127^

*Sik1/2/3* single or double knockin (KI) mice with SIK1-T182A, SIK2-T175A, or SIK3-T163A kinase inactive mutation created by conventional ES cells gene targeting technologies have contributed to the macrophage polarization.^133^ In addition, compared with counterpart mice, inactive SIK-KI mice significantly increased the production of IL-10, accompanied by the decreased IL-6 and TNFα.^133^ Similar results including inhibition of proinflammatory cytokines and promotion of LPS-induced endotoxic shock have been also observed in *Sik3* KO mice.^89^

Recently, SIK cancer-related mouse models have been reported. Hollstein et al.^42^ generated conditional floxed Kras (*Kras*^LSLG12D/+^;*R26*^LSL;luc/luc^); *Sik1*^fl/fl^ (KSik1) mouse model and combined KSik1 model with pSECC-mediated inactivation of SIK3 (KSik1 + sgSik3), which uses *Cre* recombinase to activate *Kras* and inactivate *Sik1*, and simultaneously delivers *Cas9* and a sgRNA targeting *Sik3*. These mouse models are used in the research of NSCLC, showing a more tumorigenic phenotype, including increased tumor size and burden.^42^ Another research reported that *KT*;*H11*^*LSL-Cas9*^ (*Kras*^*LSL-G12D/+*^;*R26*^*LSL-Tomato*^*;H11*^*LSL-Cas9*^) mice with each double or triple Lenti-sgRNA/Cre vector were applied to NSCLC research.^43^ They found *KT*;*H11*^*LSL-Cas9*^ mice with sgSIK1/3 displayed larger tumor size.

In addition, *Sik* knockout mouse models have also been used for other physiological process studies. For example, *Sik1*^−/−^, but not the *Sik1*^−/+^ and counterpart mice showed high blood pressure under high salt feeding conditions.^81^ Meanwhile, the increased left ventricular wall thickness caused by a high salt diet only occurred in *Sik2*^*+/+*^, but not in *Sik2*^−*/*−^ mice on a high salt diet.^134^ Interestingly, *Sik2* KO mice could enhance neuronal survival due to the potent tolerance on oxygen–glucose deprivation and transient focal ischemia.^27^ *Sik3* KO mice also displayed abnormal circadian rhythms, including phase-delayed, cycle prolongation, interference with light dark cycle, the phase change of exercise activities, and abnormal physiological rhythms with an unidentified mechanism.^135^ Based on the previous findings, the deficiency in *Siks*, especially in *Sik3*, could result in multiple metabolic abnormalities in diverse mouse organs, but SIK cancer-related mouse models have not been well-reported yet. To further distinguish the potential roles of SIK family members, especially in tumorigenesis, more tissue specific KO or functionally KI mice models will be generated under different genetic backgrounds.

## SIK functions in cancers

Although the profound functions of SIKs have been link to metabolic process regulations, accumulating studies have indicated that SIKs also play pilot roles in tumorigenesis as oncogene or tumor suppressors. Some genetic alterations, including KRAS^G12D^, GNAS^R201C^, and *Lkb1*-deficient converge to SIKs in solid tumors.^42,43,136^ The underlying mechanism and clinical relevance will be briefly summarized as below (Table 2).

**Table 2:** The functions and clinical relevance of SIK in tumorigenesis

SIK members	Tumors	Functions	Mechanisms	References
SIK1	Brest cancer	Tumor suppressor	Promote the anoikis of cancer cells through LKB1-SIK1-p53 and inhibit metastasis	^15,16^
SIK1	Ovarian cancer	Tumor suppressor	Inhibit proliferation and loss the characteristics of tumor stem cells	^137^
SIK1/3	Lung cancer	Tumor suppressor	Couple with LKB1 inhibit proliferation via IL-6 and CRTC	^42,43^
SIK1	Pancreatic cancer	Tumor suppressor	Inhibit cancer metabolic reprograming	^136^
SIK1	Pancreatic ductal cancer	Tumor suppressor	Reduce gemcitabine resistance	^138^
SIK1	Hepatocellular carcinoma	Tumor suppressor	Delay proliferation and EMT via inhibiting Wnt/β-catenin signal activation	^14^
SIK1	Lung cancer	Tumor suppressor	Inhibit EMT and increase radiation therapy	^139^
SIK1	Colorectal cancer	Tumor suppressor	Inhibit proliferation and migration	^140^
SIK2	Ovarian cancer	Oncogene	Inhibit cell apoptosis and promotes G1/S transformation by regulating centrosome, promote metastasis via boosting EMT and associates PI3K/Akt signaling pathway	^7,10,11,93,145^
SIK2	Prostate cancer	Oncogene	Regulate the cell cycle regulators p21, p27 and Cyclin D	^6^
SIK2	Diffuse large B cell lymphoma	Oncogene	Regulate glucose metabolism	^12^
SIK2	Triple negative breast cancer	Oncogene	Inhibit autophagy flux	^146^
SIK2	Breast cancer	Tumor suppressor	Inhibit the proliferation and migration and Akt activity	^147^
SIK3	Ovarian cancer	Oncogene	Couple with promote G1/S process via upregulating cyclinD/E and downregulating p21/p27	^151^
SIK3	Breast cancer	Oncogene	Promote G1/S transformation by increasing the activity of CDK2, enhance migration via upregulating CXCR4 and regulate tumor inflammation microenvironment	^148^
SIK2/3	Acute myeloid leukemia	Oncogene	Promote proliferation via SIK-HDAC-MEF2C	^150^
SIK3	Ovarian cancer	Tumor suppressor	Low expression associates with poor prognosis and resistance topaclitaxel and cisplatin	^152^

### SIK1 in cancers

**Fig. 3:**
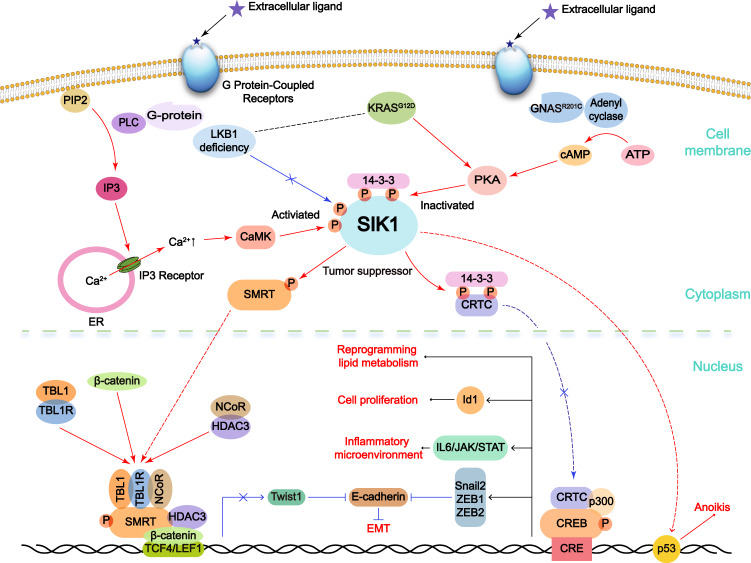
The function of SIK1 in tumorigenesis. SIK1 is a tumor suppressor gene, which plays an important regulatory role in *GNAS*, *KRAS* mutation and *LKB1* deletion mediated tumors. SIK1 inhibits CREB mediated transcription and affects cancer cell proliferation, metabolic reprogramming and inflammatory microenvironment. SIK1 can also promote cancer cell anoikis through LKB1-SIK1-p53 signaling pathway. In addition, SIK1 can inhibit Twist1 expression through Wnt/β-catenin signal pathway, and repress Snail2, ZEB1 and ZEB2 expression through modulating CRTC, and compromise the EMT process. It is worth noting that in the absence of LKB1, Ca^2+^ influx from ER to the cytoplasm via the G-protein/PLC/IP3 axis, could cause CaMK-mediated SIK1 phosphorylation and activation

SIK1 has been prone to act as a tumor suppressor in ovarian, lung, colorectal and breast cancers as well as pancreatic ductal adenocarcinoma and hepatocellular carcinoma (Fig. 3).^14,137–139^ SIK1 can promote cancer cell anoikis through LKB1-SIK1-p53 signaling pathway in breast cancer.^15,16^ Moreover, depletion of *SIK1* contributed to breast cancer distal metastasis, and low SIK1 expression is associated with poor prognosis in breast cancer patients.^15^ In keeping with this finding, overexpression of SIK1 reduced the proliferation and tumor stem cell formation of ovarian cancer.^137^ SIK1/3 have also been identified to be essential for inhibiting tumor development in a KRAS^G12D^-induced lung adenocarcinoma mouse model,^42,43^ at least partially by inhibiting IL-6-janus kinase (JAK)-signal transducer and activator of transcription (STAT) signal.^42^ Since metabolic reprogramming includes increased glycolysis and abnormal lipid metabolism, a common feature of cancer, SIKs can affect FAO to inhibit G-protein α-subunit (GNAS)-mediated extensive reprogramming of lipid metabolism and facilitate pancreatic tumorigenesis.^136^ Besides, another research observed that loss of *SIK1* is associated with gemcitabine resistance in pancreatic ductal adenocarcinoma.^138^

In the process of invasion and metastasis, tumor cells undergo epithelial–mesenchymal transition (EMT) and obtain a more aggressive phenotype. A recent study has demonstrated that SIK1 is lower expressed in hepatocellular carcinoma, and could delay hepatocellular carcinoma cell proliferation and EMT by inhibiting the Wnt/β-catenin signal.^14^ More interestingly, high levels of E-cadherin and zonula occludens-1 (ZO-1) were detected in the condition of SIK1 ectopic expression, while the silence of *SIK1* downregulated these proteins.^14^ Mechanistically, SIK1 promotes E-cadherin expression by negatively regulating the expression of its transcriptional inhibitors, such as Snail2, zinc-finger E-box binding homeobox (ZEB) 1 and ZEB2, thus blocking the EMT process.^13^ Meanwhile, the inactivation of SIK1 reduced ZEB1 expression and contributed to the invasion and migration capability of non-small cell lung cancer (NSCLC) with an anti-radiation therapy phenotype.^139^ On the other hand, SIK1 also directly phosphorylated silencing mediator like retinoid and thyroid-hormone receptors (SMRT), which would be transported to the nucleus and inhibit Twist1 expression. Specifically, Twist1 can transcriptionally inhibit SIK1 expression, thus forming a negative feedback regulatory loop between SIK1 and Twist to affect the EMT process.^14^

Recently, several MicroRNAs have been reported to target *SIK1* in promoting cancer cell proliferation or migration. Among which, miR-141 was observed to inhibit the tumor suppressive function of SIK1 and promote ovarian cancer cell proliferation.^137^ Similarly, miR17 attenuated SIK1 levels, leading to promoting colorectal cancer proliferation and migration.^140^ MiR-203^138^ and miR-373^141^ promoted pancreatic cancer and melanoma cell proliferation, migration, and invasion by degrading SIK1. As expected, lncRNAs are also acting as upstream regulators of SIK1, possibly via regulating according miRNAs. LncRNA ENST01108 served as a sponge to negatively regulate miR-489 levels, which negatively regulated *SIK1*, as a result, ENST01108 can promote glioma tumorigenesis.^142^ Analogously, lncRNA NR2F1-AS1 regulated the miR-17/SIK1 axis and inhibited the invasion and migration capability of cervical squamous cell carcinoma.^143^ In contrast, lncRNA TCONS_00029157, also termed SIK1-LNC, was positively associated with SIK1 expression, and they together inhibited lung cancer cell malignant phenotypes.^144^

### SIK2 in cancers

**Fig. 4:**
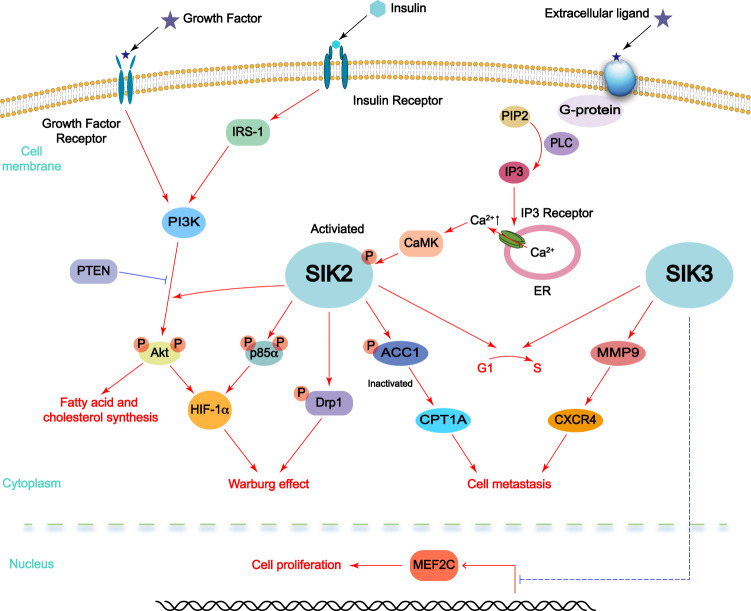
The function of SIK2 and SIK3 in tumorigenesis. SIK2 and SIK3 are regarded as potential oncogenes. SIK2 acts as an activator of PI3K/AKT signal, which promotes the Warburg effect and tumorigenesis. SIK2 also affects metabolic reprogramming, including FAO and mitochondria oxidative phosphorylation in a PI3K/AKT independent manner, and promotes tumor cell proliferation and metastasis. SIK3 boosts MEF2C-mediated tumor cell proliferation by inhibiting HDAC. In addition, SIK3 promotes tumor cell migration and metastasis through MMP9/CXCR4 axis

The role of SIK2 in tumors has been studied more extensively than that of SIK1 and SIK3. Since high expression of SIK2 appears in various cancers due to an amplified region in the chromosome 11q23, SIK2 is considered as a potential oncogenic marker for ovarian and prostate cancers, as well as glioma and diffuse large B-cell lymphoma (DLBCL).^6,10–12^ To date, SIK2 has been proved to promote tumorigenesis by modulating many aspects of cancer hallmarks (Fig. 4). For example, SIK2 could inhibit cell apoptosis and promote G1/S transformation in ovarian cancer.^11^ SIK2 phosphorylated and subsequently translocated the centrosome linker protein, c-Nap1, resulting in its cytoplasm residence and promoting the loss of centriole cohesion.^145^ In keeping with these findings, SIK2 inhibitors, such as ARN-3236, can uncouple the centrosome from the nucleus in the interphase, and attenuate the separation of the centrosome during mitosis, resulting in cell cycle arrest, cell apoptosis, and tetraploid in ovarian cancer.^10^ In prostate cancer, *SIK2* KO not only repressed the cell apoptosis through CREB-mediated ER stress response, but also led to G1 arrest by modulating the cell cycle regulators, such as p21, p27, and cyclin D/E.^6^ On the other hand, SIK2 could regulate glucose metabolism in DLBCL cell line via manipulating the CRTC–CREB axis, potentially leading to cancer progression, while high expression of SIK2 was not detectable in the primary DLBCL cells.^12^ Although the function seems to promote triple negative breast cancer growth by inhibiting the autophagy flux,^146^ SIK2 also played an active role in the process of autophagy, which can promote autophagy maturation.^9^

Total difference from SIK1 and SIK2 is prone to facilitate tumor metastasis by promoting the EMT process and enhancing the ability of tumor cell migration.^11^ *Sik2* KO compromised the metastasis of omentum and mesentery of ovarian cancer in mouse models.^11^ As we mentioned above, SIK2 can enhance fatty acid oxidation through phosphorylating ACC and augment adipocytes-induced ovarian cancer metastasis,^7^ SIK2 high expression enhanced ovarian cancer cell intraperitoneal metastasis, while *SIK2* absence prevented ovarian metastasis in vivo.^7,11^ SIK2 is also demonstrated to regulate the AKT signaling pathway, one of the most dysregulated pathways in cancers.^7,10,11,30,93^ SIK2 can directly phosphorylate p85α, a regulatory subunit of PI3K complex, to activate PI3K, contributing to ovarian cancer cell proliferation.^7^ As a result, SIK2 inhibitors could repress AKT phosphorylation and inhibit its kinase activity.^10^ Notably, a recent study showed that SIK2 promoted cancer cell glycolysis and Warburg effect via dictating PI3K/AKT/HIF1α signaling pathway, to promote ovarian cancer cell growth and metastasis.^11^ Importantly, SIK2-mediated Drp1 phosphorylation could promote mitochondrial fission to inhibit mitochondria oxidative phosphorylation.^11^ Furthermore, SIK2 also has been found to enhance fatty acid and cholesterol synthesis through upregulating the expression of SREBP1c/FASN and SREBP2/HMGCR via activating the AKT kinase, afterward promoting the proliferation of ovarian cancer cells.^93^ The absence of *SIK2* enhances the sensitivity of ovarian cancer to paclitaxel through inhibiting centrosome separation and AKT/survivin signal.^10,17,145^

Unexpectedly, SIK2 also displays a kind of tumor suppressive role, for example, some observations show that *SIK2* gene is located in the common deletion region among breast cancer,^6^ and observed that SIK2 low expression was associated with the good prognosis of breast cancer patients.^147^ Biologically, SIK2 can inhibit the proliferation and survival of breast cancer cells possibly by repressing the PI3K/AKT and RAS/ERK signaling pathways and blocking the EMT process.^147^ Taken together, accumulating studies suggest that SIK2 acts as an oncogene, and its ablation results in G1 arrest, centrosome separation inhibition, AKT kinase inactivation and EMT blockage, so targeting SIK2 may be a potential strategy for cancer therapies.

### SIK3 in cancers

SIK3 is highly expressed in around 55% breast cancer patients, and markedly governs G1/S process through upregulating the gene expression of cyclin D and cyclin E, simultaneously downregulating the expression of p21 and p27,^17^ or increasing the cyclin dependent kinase 2 (CDK2) activity (Fig. 4).^148^ The absence of *Sik3* leads to the prolongation of mitosis in mice and human cells, thus increasing the sensitivity of cancer cells to a variety of anti-mitotic drugs, including inhibition of microtubules, kinesin, and mitotic kinases.^149^ SIK3 also plays a positive role in mediating the high salt-induced inflammatory signal response that leads to cancer cell proliferation.^148^ SIK3 induces the upregulation of inflammatory arginine metabolism factors, such as iNOS and ass-1, and the downregulation of anti-inflammatory enzymes, such as arginase-1 and ornithine decarboxylase in breast cancer.^148^ Notably, ectopic expression of SIK3 in breast cancer cell lines increases matrix metalloproteinase 9 (MMP9)-C-X-C motif chemokine receptor 4 (CXCR4) signal and further contributes to cancer cell migration.^148^ Additionally, SIK3 elevated the transcriptional activity of myocyte enhancer factor 2C (MEF2C) by inhibiting HDAC4 catalytic functions to accelerate acute myeloid leukemia progression.^150^ On the other hand, SIK3 is also considered as an oncogene and ovarian cancer tumor-associated antigen,^151^ however, low SIK3 expression is also linked to poor overall survival (OS) and progression free survival (PFS) in advanced serous ovarian cancer. The *SIK3* KO ovarian cancer cells display resistance to paclitaxel and cisplatin-mediated chemo-therapies by enhancing the binding with ATP cassette subfamily G member 2 (ABCG2), a transporter for drug efflux.^152^

## Potential inhibitors targeting SIKs

**Fig. 5:**
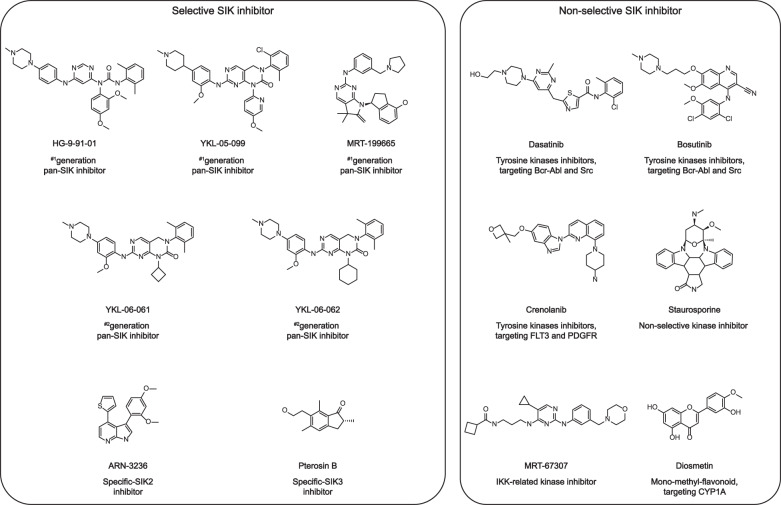
Potential inhibitors targeting SIKs. SIK inhibitors are divided into selective and non-selective inhibitors. HG-9-91-01, YKL-05-099, MRT-199665, YKL-06-061, and YKL-06-062 are all selective pan-SIK inhibitors. ARN-3236 and pterosin B inhibit SIK2 and SIK3, respectively. Dasatinib, bosutinib, staurosporine, diosmetin, crenolanib and MRT-67307 have non-selective inhibitory effects on SIKs by targeting different kianses labled as below

In recent years, numerous efforts have been devoted to developing SIK inhibitors, especially to target the oncogenic SIK kinase (Fig. 5). HG-9-91-01 is an effective and relatively selective SIK inhibitor, which can target all the SIK proteins to regulate their biological functions, such as gluconeogenesis and secretion of pro-inflammatory factors.^8,33,133,153–155^ HG-9-91-01 is working not only by occupying the ATP-binding sites, but also by binding a small hydrophobic vesicle near this site.^8^ YKL-05-099, another SIK inhibitor, was derived from HG-9-91-01 with improved selectivity on SIK1 and SIK3, but it also can inhibit other tyrosine kinases, such as Brk and Lck.^29,155^ Treated with YKL-05-099, SIK functions were largely restricted especially by increased IL-10, decreased IL-6 and TNFα, however, there is no obvious effect on the metabolism in mice.^29,155^ This inhibitor can also rapidly inhibit MEF2C function by targeting SIK3 and diminish the phosphorylation and nuclear localization of HDAC4. YKL-05-099 can also alleviate the disease progression in vivo and prolong the survival of the animals at a well-tolerated dose upon treating two different MLL-AF9 acute myeloid leukemia mouse models.^156^ It was also reported that a single point mutation of *SIK3* (T142Q) or the inactivation of HDAC4 were enough to acquire the resistance to the YKL-05-099 treatment.^156^

MRT-199665 is developed as an effective, ATP competitive, selective MARK/SIK/AMPK inhibitor.^8^ MRT-199665 inhibits SIK-mediated CRTC3 phosphorylation to increase LPS-stimulated IL-10 production and inhibit the secretion of proinflammatory cytokines, such as IL-6, IL-12, and TNFα in macrophages.^8,85^ MRT-199665 can also induce apoptosis of human acute myeloid leukemia cells by activating MEF2C in vitro,^157^ and enhance TGF-β-mediated apoptosis and death of murine mammary epithelial cells.^85^ YKL-06-061 and YKL-06-062 are employed as selective second-generation inhibitors of SIKs. The structures of these two inhibitors are analogous, and the treatment of these inhibitors results in an increase of *MITF* mRNA expression in a dose-dependent manner.^158^

ARN-3236 is another relatively selective SIK2 inhibitor (IC_50_ < 1 nM) with oral activity,^10,71^ which can prevent the centrosome separation in the mitotic process, leading to ovarian tumor cell sensitizing to paclitaxel treatment.^10^ Thus, it is suggested that SIK2 inhibitors can be used in combination with paclitaxel for ovarian cancer therapies.^145^ Pterosin B, an indanone found in pteridium aquilinum, is a kind of specific inhibitor of SIK3.^102,132^ Although pterosin B cannot directly inhibit SIK3 kinase activity, it can promote the interaction between SIK3 and the glycogen phosphorylase kinase gamma subunit (PHKG2), a CaMK family kinase for increasing the self-inhibition of SIK3 and leading to inhibiting SIK3 downstream cascades.^102^ Hence, pterosin B can further promote glucose production by up regulating gluconeogenic expression and reducing glycogen content in mouse hepatoma AML-12 cells.^102^ Interestingly, intraarticular injection of pterosin B can inhibit chondrocyte hypertrophy and protect cartilage from osteoarthritis via inhibiting SIK3 kinase.^132^

Compared with other members of the AMPK family, SIKs have a small threonine residue at the “gatekeeper” site,^120^ thus SIKs and some tyrosine kinases share a similar structure on the kinase domain. As a result, several clinically approved drugs for antagonizing tyrosine kinases, such as Bcr-Abl and Src, potentially inhibit SIK kinase activity and oncogenic functions. For example, bosutinib and dasatinib were initially found to inhibit tyrosine kinase of Bcr-Abl, Src and Tec family members for the treatment of chronic myeloid leukemia.^159^ Similar to the pan-SIK inhibitor HG-9-91-01, dasatinib and bosutinib exhibit notably inhibitory function on SIK kinase activity and regulate macrophage polarization.^120,154^ Furthermore, dasatinib and bosutinib can promote TGFβ-mediated apoptosis by repressing SIK kinase activity in vitro.^85^ In addition, bosutinib can also inhibit SIK by blocking the Cdc37–Hsp90 chaperone system and lead to their ubiquitylation and degradation.^91,102^ Crenolanib is an effective and selective III receptor tyrosine kinase inhibitor, targeting FLT3 and PDGFR.^160,161^ Crenolanib has been found to have a strong non-targeting effect on SIK (IC_50_ in vivo for SIK2 is 16 nM, for SIK3 is 2 nM), and shows good tolerance in human patients.^156^

Staurosporine is likely to inhibit both SIK1 and SIK2 functions, and further considered as a nonselective kinase inhibitor at high concentrations to repress many kinases, including PKC, CaMK.^162,163^ Staurosporine also increases CRTC2 abundance in nucleus and triggers CRH transcription in 4B cells by repressing SIKs.^162^ MRT-67307 was originally used to inhibit IKK-related kinase, now MRT-67307 has been employed to inhibit SIKs, like HG-9-91-01, to increase the production of IL-10 and notably to inhibit the secretion of pro-inflammatory cytokines.^8,164^ Mono-methyl-flavonoids, such as diosmetin (4′-O-metlylluteolin), have also been revealed to effectively inhibit SIK2 kinase activity and promote the nuclear translocation of CRTC1.^125^ But this effect is nonselective, and diosmetin has also been found to inhibit other enzymes, such as CYP1A.^165,166^ Taken together, up to date, the studies of SIK specific inhibitors are mainly focusing on their effects on metabolism and inflammation, however, few researches have evaluated their roles in tumorigenesis, even in the clinical trial, which will warrant to be further investigated.

## Discussion and perspective

It is generally accepted that SIKs are important regulators involved in many metabolic processes. However, as we described above, the role of SIKs in tumorigenesis is more complex and controversial. Of note, SIK1, acting as a tumor suppressor, can inhibit the EMT process resulting in reducing cancer metastasis and promoting cancer apoptosis.^13–16^ By contrast, SIK2, serving as an oncogene, positively regulates cell proliferation and apoptosis by governing cell cycle and autophagy.^6,9–12^ SIK2 can also regulate the glycolipid metabolism of tumor cells and promote the Warburg effect partially via increasing the AKT kinase activity.^11^ SIK3 is playing a potential oncogenic role and positively regulates the G1/S process to promote breast and ovarian cancer cell proliferation.^17,148,151^ As a result, SIK family members play distinct roles in the context of different cancer types. This may be due to the lack of understanding of the upstream regulators and downstream effectors of SIKs. Nevertheless, the *SIK* inactivation mutations are not frequently occurred in human cancers, which may partially reflect the redundant roles of SIKs in tumorigenesis.^136^

Here, we have comprehensively summarized the structure, upstream regulators and downstream effectors of SIKs, as well as their potential roles under physiological and pathological conditions, especially in tumorigenesis. However, there are emerging questions need to be paid more attention and well investigated in the future. First, the diverse regulations of SIKs in genomics, epigenetics, and PTM levels are rarely studied. Second, unlike CRTC and class IIa HDACs, two important and well-investigated SIK substrates, the relationship between SIKs and TGFβ-Smad, AKT, Hippo, and NF-κb pathways have not been clearly evaluated yet. Third, the relationship between SIKs and tumor is still controversial. The roles of SIKs-mediated energy metabolism and inflammation regulation in tumorigenesis have not been well illustrated. Fourth, these three SIK family members have shared similar structures, but they play the same or different functions in physiological and pathological processes. Therefore, the redundancy and difference of different SIK family members function desired for further exploration. Fifth, *Sik-*associated KO or KI mouse models, especially the conditional KO mouse models would be generated to further explore the potential roles of SIKs in tumorigenesis or other diseases. Lastly, more specific and effective small molecule inhibitors targeting SIKs need to be developed, and their potential effects in diseases, especially in tumors, need to be explored in both in vitro and in vivo experiments. Collectively, there is no doubt that SIKs play an important role in tumor cell proliferation, apoptosis, survival, and metastasis by regulating multiple processes including the metabolic homeostasis and inflammation, which highlights the potential strategy to target SIK for cancer therapies in the near future.
